# Assessment of Cutting-Balloon Angioplasty with Novel Bioabsorbable Polymer-Coated Everolimus-Eluting Stent in Treating Calcified Coronary Lesions Guided by Intravascular Ultrasound (CUPID Trial): study design and protocol

**DOI:** 10.1186/s13063-024-08484-0

**Published:** 2024-10-29

**Authors:** Jihun Ahn, HyeYon Yu, Sangho Park, Jon Suh

**Affiliations:** 1 Department of Internal Medicine, Daejeon Eulji Medical Center, Eulji University School of Medicine, Daejeon, Korea; 2 Department of Nursing, College of Medicine, Soonchunhyang University, Asan, Korea; 3 Department of Internal Medicine, Soonchunhyang University Cheonan Hospital, Soonchunhyang University College of Medicine, Cheonan, Korea; 4 Department of Internal Medicine, Soonchunhyang University Bucheon Hospital, Soonchunhyang University College of Medicine, Bucheon, Korea

**Keywords:** Cutting balloon, Calcification, Percutaneous coronary intervention

## Abstract

**Background:**

Various devices and techniques have been used for plaque modification in the treatment of severe coronary artery calcification. This prospective, multicenter, randomized, open-label study aims to evaluate the safety and efficacy of cutting balloon angioplasty using a novel bioabsorbable polymer-coated everolimus-eluting coronary stent for treating various degrees of calcified coronary lesions.

**Methods:**

We outline the trial design aimed at assessing whether the cutting balloon (Wolverine™) is non-inferior to the non-compliant balloon in treating patients with calcified lesions, encompassing both de novo and in-stent restenosis (ISR) lesions. We aim to enroll 250 patients who have undergone bioabsorbable polymer-coated everolimus-eluting coronary stent (Synergy™) implantation. The primary endpoint is the minimal stent cross-sectional area at the calcium site as determined by intravascular ultrasonography. The secondary endpoints include major adverse cardiac events and target lesion revascularization at 12 months, alongside procedural convenience and operator-centric parameters, such as the number of balloons used, procedure time, and total contrast medium volume used.

**Discussion:**

In this study, we will evaluate the efficacy and safety of Wolverine™ and non-compliant balloon in patients with calcified coronary lesions and provide a rationale for which type of balloons will optimally modify calcium lesions. In addition, we will attempt to expand the indications of the cutting balloon for treating mild-to-severe calcified coronary lesions. As the scope of insurance coverage for cutting balloons remains limited in some countries, this study may provide evidence for extending insurance coverage to the treatment of de novo calcified and ISR lesions.

**Trial registration:**

ClinicalTrials.gov NCT06177808. Registered on January 1, 2024.

## Administrative information

**Table:** 

Title {1}	Assessment of Cutting-Balloon Angioplasty with Novel Bioabsorbable Polymer-Coated Everolimus-Eluting Stent in Treating Calcified Coronary Lesions Guided by Intravascular Ultrasound (CUPID Trial): study design and protocol
Trial registration {2a and 2b}	ClinicalTrials.gov ID: NCT06177808
Protocol version {3}	Protocol number CUPID version 1.2
Funding {4}	Boston Scientific Korea: There are no roles in the design of the study, the collection, analysis, and interpretation of data, or in writing the manuscript
Author details {5a}	Jihun Ahn: Department of Internal Medicine, Daejeon Eulji Medical Center, Eulji University School of Medicine, Daejeon, Korea HyeYon Yu: Department of Nursing, College of Medicine, Soonchunhyang University, Asan, Korea Sangho Park: Department of Internal Medicine, Soonchunhyang University Cheonan Hospital, Soonchunhyang University College of Medicine, Cheonan, Korea Jon Suh: Department of Internal Medicine, Soonchunhyang University Bucheon Hospital, Soonchunhyang University College of Medicine, Bucheon, Korea
Name and contact information for the trial sponsor {5b}	Jon Suh E-mail: immanuel@schmc.ac.kr
Role of sponsor {5c}	Sponsor: Soonchunhyang University Hospital Responsible party: principal investigator Investigator: Jon Suh [jsuh] Official title: Jon Suh, MD/PhD, principal investigator Affiliation: Soonchunhyang University Hospital Collaborators: Boston Scientific Corporation Soonchunhyang University Hospital Eulji University The Catholic University of Korea Hanil General Hospital, Korea

## Introduction

### Background and rationale {6a}

Significant calcification of coronary artery lesions remains a challenging condition and is associated with stent delivery failure and poor clinical outcomes [1, 2]. Heavily calcified lesions respond poorly to conventional balloon angioplasty, particularly when stents are implanted within incompletely modified lesions, leading to incomplete and asymmetrical stent expansion [3, 4]. Insufficient calcium levels and inadequate plaque modifications are associated with poor clinical outcomes [5]. Moreover, incomplete modification and preparation of calcified lesions adversely affect various procedural aspects, including the number of balloons used, amount of contrast medium used, and procedure duration, causing stent delivery failure [6]. Periprocedural factors, such as contrast medium volume and procedure duration, are also recognized as contributors to increased adverse clinical outcomes and in-hospital complication rates [7, 8]. Various devices, such as cutting balloons, spiral balloons, and rotational atherectomy devices, have been used to modify severely calcified coronary lesions [9, 10].

The cutting balloon has four surrounding blades; upon inflation, these blades create incisions in the plaque. Subsequently, the shear force exerted by the balloon inflation propagates the cracks, resulting in lumen enlargement [11]. However, there is limited evidence regarding the use of the cutting balloon in treating various degrees of calcified lesions including in-stent restenosis (ISR). In addition, despite the advantages of using the cutting balloon in treating calcium lesions, studies specifically designed to examine intraprocedural and operator-oriented endpoints, such as the number of balloons used, procedure duration, and contrast medium volume used, are scarce. Moreover, bioabsorbable polymer-coated coronary stents have shown safety and efficacy in treating coronary artery diseases, including calcified lesions [12].

### Objectives {7}

In this study, we aim to evaluate the safety and efficacy, encompassing both intraprocedural and operator-oriented outcomes, of a novel cutting balloon (Wolverine™, Boston Scientific, Natick, MA, USA) in comparison with a non-compliant (NC) balloon for the treatment of patients with various degrees—from mild to heavily—calcified lesions. The patients will undergo intravascular ultrasound (IVUS)-guided bioabsorbable polymer-coated everolimus-eluting coronary stents (Synergy™, Boston Scientific Corporation, Marlborough, MA, USA) implantation.

### Trial design {8}

**Fig. 1:**
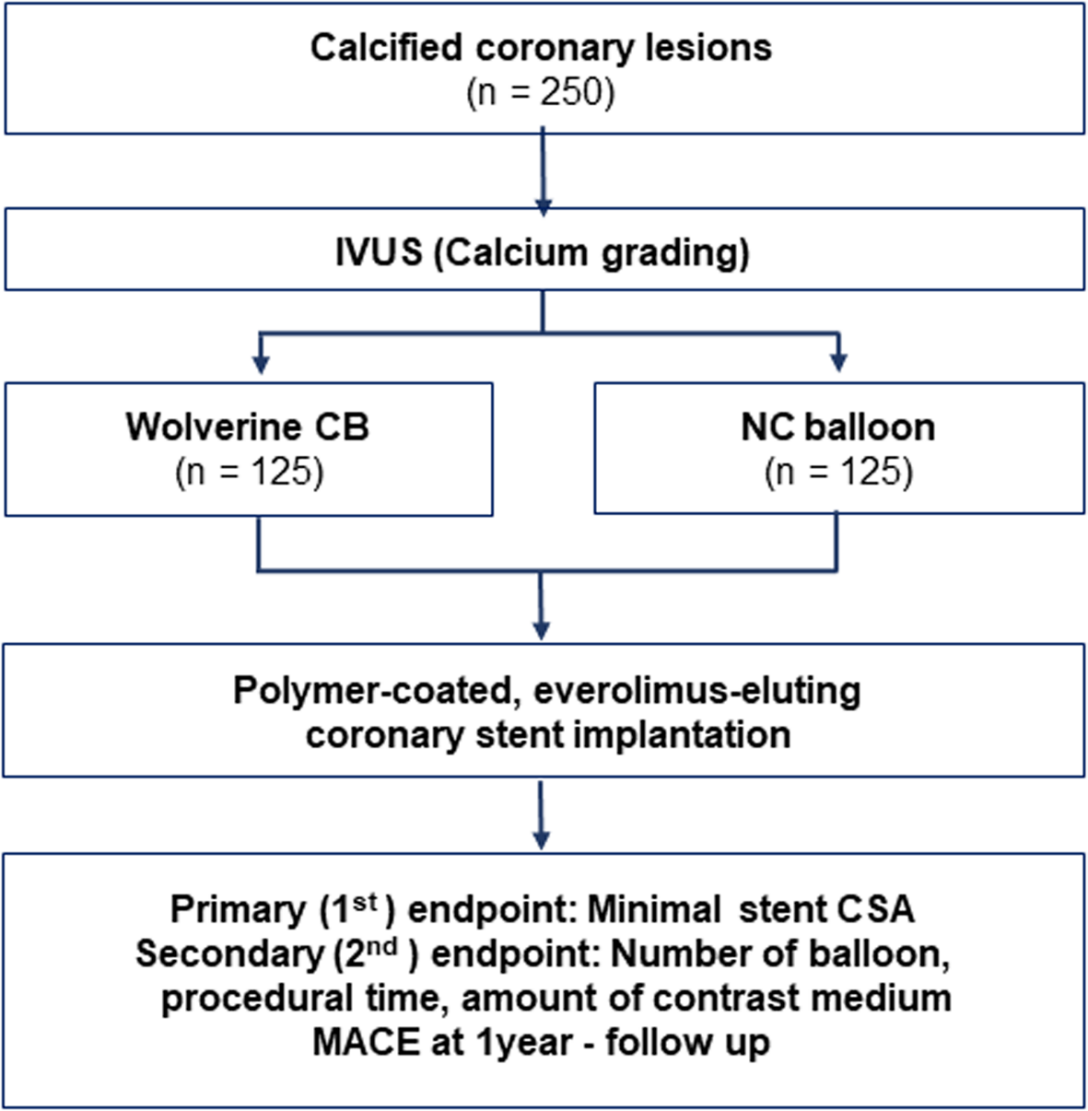
Study design overview. Patients with significant stenosis observed in coronary angiography with calcified lesions undergo IVUS if they meet the enrollment criteria. After randomization into the Wolverine CB group and the NC balloon group, patients undergo the procedure. Subsequent to sufficient balloon dilation (Wolverine CB vs. NC balloon), a polymer-coated, everolimus-eluting coronary stent is implanted. After the index procedure, the primary and secondary endpoints are evaluated, and a 1-year follow-up is conducted for the occurrence of MACE, one of the secondary endpoints

This prospective multicenter randomized open-label trial will explore the non-inferiority of cutting balloon pre-dilatation in comparison with NC balloon pre-dilatation for the treatment of calcified lesions in patients undergoing coronary stent implantation (Fig. 1).

## Methods: participants, interventions, and outcomes

### Study setting {9}

This prospective multicenter randomized open-label trial will be conducted across multiple centers. The following devices will be used: (1) a Wolverine™ (Boston Scientific, Natick, MA, USA) cutting balloon, (2) a bioabsorbable polymer-coated everolimus-eluting coronary stent (Synergy™, Boston Scientific Corporation, Marlborough, MA, USA), and (3) an NC balloon (the specific type of NC balloon will be determined at the operator’s discretion).

### Eligibility criteria {10}

#### Inclusion criteria

1. Patients with coronary artery disease including ISR or de novo lesion with ≥ 70% diameter stenosis
2. Target-lesion calcification confirmed via imaging (an arch of calcium of at least 100°)
3. Reference vessel diameters between 2.0 and 4.0 mm considered suitable for percutaneous coronary intervention (PCI)

#### Exclusion criteria

1. Extremely tortuous or angulated lesions
2. Lesions with dissection before balloon pre-dilatation
3. Lesions within a vein graft
4. Extremely narrow lesions in which IVUS cannot pass even after pre-dilatation with a cutting or NC balloon
5. ST-elevation myocardial infarction
6. Comorbidities that preclude the achievement of a year follow-up

### Who will take informed consent? {26a}

The investigators will obtain informed consent from all participants or legal guardians before the PCI.

### Additional consent provisions for collection and use of participant data and biological specimens {26b}

No additional consent provisions are specified for the collection and use of participant data and biological specimens. However, if ancillary studies involving the collection and use of participant data and biological specimens are conducted, additional consent provisions will be implemented.

## Interventions

### Explanation for the choice of comparators {6b}

The choice of comparators—Wolverine cutting balloons, NC balloons, and bioabsorbable polymer-coated everolimus-eluting coronary stents—was based on their widespread application in PCI procedures for treating calcified lesions and plaque modification.

### Intervention description {11a}

#### Medication and procedural techniques

Participants will be randomly assigned to undergo pre-dilatation with either a Wolverine cutting balloon or an NC balloon before stent implantation. Prior to PCI, all patients will be administered loading doses of aspirin (200–300 mg), clopidogrel (300–600 mg), ticagrelor (180 mg), or prasugrel (60 mg). After sheath insertion at the arterial access site, unfractionated heparin will be administered at a bolus dose of 100 U/kg, adjusted for the patient’s weight. Additional boluses will be administered to maintain an activated clotting time of 250–300 s. Additional intravenous and intracoronary injections of platelet glycoprotein IIb/IIIa receptor blockers will be provided at the operator’s discretion. The interventional strategy, as well as the sizes of the balloon and stent, will be determined by the same operator for all patients. Following randomization, pre-dilatation will be conducted using either a Wolverine or NC balloon followed by the deployment of a synergy stent. If the lesions prove challenging for balloon crossing, atheroma modification via rota-ablation will be performed at the operator’s discretion, and such cases will be excluded from enrollment. All patients undergoing PCI will be prescribed aspirin (100 mg daily) with clopidogrel (75 mg daily), ticagrelor (180 mg), or prasugrel (60 mg) as per the recommendation for a dual antiplatelet maintenance regimen for at least 12 months. Cilostazol (100–200 mg daily) will be administered as an adjunct to the dual antiplatelet regimen at the treating physician’s discretion. During the in-hospital stay and post-discharge, patients will be prescribed medications including aspirin, clopidogrel, ticagrelor, prasugrel, beta blockers, calcium channel blockers, angiotensin-converting enzyme inhibitors, angiotensin receptor blockers, and lipid-lowering agents such as statins. These medications will be recorded and entered into a dedicated computerized database with clinical information.

#### Quantitative coronary angiography

The results of coronary angiography will be digitally recorded at baseline and immediately after the procedure (post-ballooning and post-stenting) and subsequently assessed offline in the angiographic core laboratory. Independent interventional cardiologists blinded to the type of balloon used, treatment outcomes, and IVUS analysis will perform the quantitative coronary angiographic analysis using an automated edge-detection algorithm (CAAS-5; Pie-Medical, Best, Netherlands) with conventional methods. All measurements will be performed using cineangiograms recorded after intracoronary nitroglycerin injection. The interpolated reference diameter will serve as the reference segment diameter. Lesion length will be defined as the distance from the proximal shoulder to the distal shoulder of the lesion. Standard qualitative and quantitative analyses and definitions will be applied for quantitative coronary angiography (QCA) [13, 14].

#### IVUS procedure and analysis

After standard coronary angiography, IVUS will be performed using OptiCross 60 MHz (Scientific Corporation, Maple Grove, MN, USA) at baseline (before pre-dilatation) and repeated immediately after stent implantation in all cases. Following intracoronary nitroglycerin injection, the IVUS catheter will be carefully advanced at least 20 mm distal to the culprit lesion under fluoroscopic guidance. Subsequently, it will be automatically withdrawn at 0.5 mm/s to initiate the imaging sequence, which will commence 20 mm distal to the culprit lesion and end at the aorto-ostial junction. Pre-dilatation of lesions that cannot be passed by the IVUS catheter will be performed using a small balloon. IVUS imaging will be recorded on a compact disc for offline analysis by a single experienced observer who is blinded to the clinical and angiographic information. IVUS measurements will adhere to the American College of Cardiology Clinical Expert Consensus Document on the Standards for Acquisition, Measurement, and Reporting of Intravascular Ultrasound Studies. The ischemia-related vessel will be identified as narrow (≥ 70%) based on angiographic findings. Calcification will be characterized as bright echoes with acoustic shadowing on IVUS, with calcification exceeding 270° considered severe. The culprit lesion site selected for analysis will be the image slice with the lowest luminal cross-sectional area (CSA). The proximal and distal reference segments will be delineated as the most normal-looking cross-sections within the same arterial segment, typically located ≤ 10 mm from the lesion but before any major (1.5 mm in diameter) side branch.

We will measure the lumen in the minimum lumen area slice. The CSA of the lumen will be measured at every 1 mm of the culprit lesion segment, and the average lumen CSA will be calculated. Additionally, we will measure the maximum calcium arc, length, and ratio (calcium length/lesion length). After implantation, the minimum stent CSA, minimum and maximum stent diameters, and acute CSA gain will be measured. Acute CSA gain is calculated as the minimum stent CSA subtracted by the minimum CSA. Stent symmetry will be assessed as the ratio of the minimum stent diameter to the maximum stent diameter. Stent expansion will be calculated as the ratio of the minimum stent CSA to the reference lumen CSA. Incomplete stent apposition will be defined as inadequate contact between the struts and the underlying wall. Stent asymmetry will be characterized by less than 70% stent symmetry in at least one section. Stent under-expansion will be defined as a stent expansion rate below 80%.

### Criteria for discontinuing or modifying allocated interventions {11b}

Participation in this study will be completely voluntary and dependent on the participant’s consent. Participants have the right to withdraw from the study at any point after their initial participation. Refusal to participate in the study or withdrawal from the study will not result in any disadvantageous treatment from the medical staff or hospital, and the participants will continue to receive the best possible care. In the event of significant reasons arising during the study that necessitate early termination or withdrawal of participation, participants will be encouraged to contact the principal investigator or research coordinator. Should participation be discontinued during the study, the research team will explore follow-up options. This process will be conducted through a separate form where participants will have various options to choose from regarding how their information will be handled upon discontinuation and whether future contact will be maintained. The reasons for discontinuing participation will be documented in the case report form (CRF) and supporting documents. Participants who discontinue their participation will not be replaced.

Reasons for deciding to discontinue participation the study may include but are not limited to the following:

- Failure to comply with instructions from research personnel
- Determination by the researcher that continuing participation would be harmful to the participant
- Pregnancy
- Need for treatment not permitted in this study
- Cancelation of the study
- Unforeseen circumstances

### Strategies to improve adherence to interventions {11c}

Strategies to improve adherence to intervention protocols include administering medications per the guidelines [15] and ensuring proper procedural techniques during PCI.

### Relevant concomitant care permitted or prohibited during the trial {11d}

Relevant concomitant care permitted during the trial includes adjunctive medications such as cilostazol, whereas prohibited care is not specified.

### Provisions for post-trial care {30}

Provisions for post-trial care will be provided, including necessary medical attention and support for any adverse events experienced by the participants during or after the trial.

### Outcomes {12}

Primary outcome: The efficacy of cutting balloons compared with NC balloons for the treatment of calcified lesions, measured by minimal stent CSA with IVUS at the calcium site.

Secondary outcomes: The comparison of clinical outcomes, such as major adverse cardiac events (MACEs) and target lesion revascularization, at 1 year. In addition, intraprocedural and periprocedural factors, such as procedure duration, the total amount of contrast medium used, and the number of balloons between the cutting balloons, will be analyzed between the two groups.

### Participant timeline {13}

During the baseline visit (V1) on day 0, the following procedures will be conducted: (1) obtaining informed consent from participants, (2) collecting demographic information, (3) recording vital signs, and (4) evaluating the inclusion/exclusion criteria. Additionally, we will also review medical history, document current medication usage, assess laboratory findings, and examine echocardiographic findings.

Approximately 1 month post-operatively (POD 1 month ± 2 weeks), the inclusion/exclusion criteria, current medication usage, and laboratory findings will be reassessed. An occurrence analysis will also be conducted at visit 2 (V2). At visit 3 (V3), approximately 6 months post-operatively (POD 6 months ± 8 weeks), similar assessments as those conducted at V2 will be performed. Similarly, approximately 12 months post-operatively (POD 12 months ± 8 weeks), assessments mirroring those conducted at V2 and V3 will be performed (visit 4, V4). At each follow-up visit, occurrence analysis will be performed to monitor events or incidents. Additionally, interventions such as coronary angiography, PCI, and VUS will be conducted as necessary at V1 (Table 1).

**Table 1:** Study visits and procedures

Study visit	Visit 1 (V1)	Visit 2 (V2)	Visit 3 (V3)	Visit 4 (V4)
Time	Day 0, baseline	1 month (± 2 weeks) post-operatively	6 months (± 8 weeks) post-operatively	12 months (± 8 weeks) post-operatively
Informed consent^a^	X			
Demographic information^b^	X			
Vital sign	X			
Inclusion/exclusion criteria	X	X	X	X
History^c^	X			
Current medication^d^	X	X	X	X
Laboratory findings^e^	X	X	X	X
Echocardiographic findings^f^	X			
Coronary angiography (CAG)	X			
Percutaneous coronary intervention (PCI)	X			
IVUS	X			
Events analysis^g^		X	X	X

^a^No procedures of this clinical trial should commence before obtaining the participant’s consent

^b^Demographic information: participant initials, sex, date of birth, smoking status, weight, height

^c^History: hypertension, diabetes, coronary artery disease, peripheral artery disease, chronic kidney disease (CKD), CKD on dialysis, heart failure, stroke, dyslipidemia, familial history of cardiovascular disease (myocardial infarction, stroke, or sudden cardiac death), current smoking

^d^Current medication (only medications used within the month before consent acquisition are investigated and recorded): antiplatelet agent, renin-angiotensin system blockade, beta blocker, calcium channel blocker, furosemide, aldosterone antagonist (including spironolactone), statins, anti-arrhythmic agent, hypoglycemic agents, anticoagulants

^e^Laboratory findings: cell blood counts (white blood cell, hemoglobin, hematocrit, platelet), blood urea nitrogen, creatinine, glucose, HbA1c (for diabetic patients), lipid panel, aspartate aminotransferase/alanine aminotransferase, total bilirubin, glomerular filtration rate, uric acid, C-reactive protein, troponin-T, creatine kinase-myoglobin binding, N-terminal pro-brain natriuretic peptide

^f^Echocardiographic findings: ejection fraction, E/e’, right ventricular systolic pressure, left atrial volume index

^g^Follow-up visits may be replaced with telephone surveys

### Sample size {14}

In this randomized controlled trial, the study sample will be matched in a 1:1 ratio, with patients being assigned to either the cutting balloon or NC balloon group. The limited clinical trials on cutting balloons for the treatment of de novo calcified coronary lesions of various degrees have produced inconsistent findings. A previous study investigating the efficacy of cutting balloons reported a mean CSA of 6.26 mm^2^ after cutting balloon treatment for native coronary artery disease in the cutting balloon group [16]. We hypothesized that stent implantation after the cutting balloon pre-dilatation would not be inferior to pre-dilatation with an NC balloon for the treatment of de novo calcified coronary lesions. Using double-sided inspection with *α* = 0.05 and *β* = 0.2, and considering an expected loss to follow-up of 20%, a minimum of 250 patients should be included. Thus, we aim to enroll 125 patients in each group. Nevertheless, the proposed sample size is 250 patients in each group to further increase power and account for possible protocol deviations.

### Recruitment {15}

Strategies will be implemented to ensure adequate participant enrollment to achieve the target sample size. Participants will be recruited from the in-patient and outpatient units of the Department of Cardiology and Internal Medicine in the participating hospitals.

## Assignment of interventions: allocation

### Sequence generation {16a}

Randomization will be conducted using a computer-generated randomization sequence, allocating participants in a 1:1 ratio to the respective treatment groups.

### Concealment mechanism {16b}

The allocation sequence will be concealed through the use of sequentially numbered, opaque, sealed envelopes.

### Implementation {16c}

After enrollment, the study will be conducted by the investigator. All participants will be followed up after undergoing PCI by each physician and investigator at the participating hospital. Each investigator at the participating centers will allocate participants in a 1:1 ratio to the respective treatment groups.

## Assignment of interventions: blinding

### Who will be blinded? {17a}

Blinding is not feasible for both patients and physicians owing to the nature of the interventions and study design; however, independent interventional cardiologists will be blinded to the type of balloon used during the outcome assessment. The data analysts will conduct the analysis of clinical outcomes, including IVUS analysis, in a blinded manner to minimize potential bias and ensure the integrity of the results.

### Procedure for unblinding, if needed {17b}

Unblinding procedures are not applicable as blinding will not be implemented in this open-label trial.

## Data collection and management

### Plans for assessment and collection of outcomes {18a}

The registration of study participants, evaluation of primary and secondary endpoints after PCI, and data collection will be conducted at each participating institution. Final data collection from each research institution and data assessment, coordination, and outcome collection will be managed at the Cardiovascular Center of Korea University Bucheon Hospital.

### Plans to promote participant retention and complete follow-up {18b}

Efforts will be made to promote participant retention through regular follow-up visits and communication, thereby ensuring the completion of the trial.

### Data management {19}

Source documents encompass electronic documents, including all records, data, and documentation containing evidence of a patient’s existence validate the integrity of the collected data. It includes hospital records, obligatory records, participant records, memos, pathology test results, participant diaries, assessment forms, pharmacy medication dispensing records, data recorded using automated testing devices, test certificates and their official copies, microfiches, microfilms, radiological examination data, audio tapes, pharmacy records, pathology laboratory records, and other documents containing evidence. We will ensure that all data entered into the electronic CRF (e-CRF) are based on and align with source documents and that any discrepancies are duly explained. Researchers may need to request previous medical records or provide current obligatory records upon request by the sponsor.

All clinical data will be collected using a web-based e-CRF via an electronic platform. Researchers and coordinators at each research institution will input and edit data through a secure network with access credentials (user IDs and password). Each researcher will have access to data from their respective institution throughout the study period. The e-CRF will be continuously updated to reflect the participants’ status at each stage during the study period. Appropriate encrypted participant numbers will be used in the e-CRF. Researchers will maintain separate confidential records (participant identification logs) to verify the identities of all registered participants. Data collected for each participant during the investigation period will be accurately and completely retained in the CRF to the fullest extent possible.

### Confidentiality {27}

Participants’ privacy and confidentiality will be upheld throughout the study. To safeguard personal data, national legal requirements regarding data confidentiality will be adhered to, including compliance with the EU General Data Protection Regulation (GDPR). In accordance with the local data protection laws, appropriate consent for the collection, use, disclosure, and/or transfer (if applicable) of personal information will be obtained. A unique participant identifier will be allocated to each participant, assigned chronologically before proceeding with study screening. Participant identifiers, rather than names, will be used to collect, store, and report participant information, including documentation, in the e-CRF. If a participant’s name appears in any other document such as a medical report, it will be redacted in the copy of the document to be uploaded to the e-CRF. The investigator is required to retain records and documents, including signed informed consent forms related to the study, for 15 years following the study completion and final publication. No records may be destroyed during the retention period without written approval from the sponsor.

### Plans for collection, laboratory evaluation, and storage of biological specimens for genetic or molecular analysis in this trial/future use {33}

No biological specimens will be collected or stored for genetic or molecular analyses.

## Statistical methods

### Statistical methods for primary and secondary outcomes {20a}

Data for all endpoints will be evaluated using an intention-to-treat analysis. Data will be presented as means and standard deviations. Continuous variables will be compared using an unpaired Student’s *t*-test or the Mann–Whitney *U* test, whereas categorical variables will be compared using the chi-squared or Fisher’s exact tests. The primary and secondary endpoints will be analyzed using both intention-to-treat (patients assigned to the treatment group) and per-protocol (patients who complete the treatment protocol) analyses. Multivariate analysis will be performed using multiple linear regression to determine the association with the minimal stent CSA. Time-to-event outcomes of MACEs will be summarized using Kaplan–Meier survival estimates, and between-group comparisons will be made using log-rank tests. *P* < 0.05 will be considered statistically significant. All statistical analyses will be performed using SPSS software (version 20.0, SPSS Inc., Chicago, IL, USA).

### Interim analyses {21b}

Interim analyses are not planned owing to the short duration of the trial.

### Methods for additional analyses (e.g., subgroup analyses) {20b}

Subgroup analyses may be conducted based on lesion characteristics, comorbidities, or procedural factors if deemed necessary.

### Methods in analysis to handle protocol non-adherence and any statistical methods to handle missing data {20c}

Multiple imputation or sensitivity analyses will be performed to handle missing data. Protocol non-adherence will be addressed through per-protocol analyses.

### Plans to give access to the full protocol, participant-level data, and statistical code {31c}

Access to the full protocol, participant-level data, and statistical codes will be provided upon request to ensure transparency and reproducibility.

## Oversight and monitoring

### Composition of the coordinating center and trial steering committee {5d}

The Executive Committee will be responsible for steering or monitoring the study. Considering the multicenter nature of the trial, the Executive Committee is comprised of the principal investigator (PI) from the main center (chairman) and investigators from the participating centers (committee members). Patients will be under constant monitoring, and any adverse events would be recorded by the investigators. In addition, monthly meetings managed by the chairman are held to maintain constant communication among the researchers and audit the trial conduct. As the PI, chairman is responsible for the design and conduct of the trial, organizing committee meetings, and publication of study results and reports. Monthly trial steering committee meetings managed by the chairman will be held to maintain constant communication among the researchers and audit the trial conduct. Day-to-day trial support will be provided by the principal investigator and cardiologists from participating centers, responsible for the trial progression, adherence to the protocol, patient safety, and consideration of new information relevant to the research question. They will oversee the administrative progress of the study and approve the final trial design and protocol issued to the Data and Safety Monitoring Board (DSMB) and clinical sites. This Executive Committee will be responsible for reviewing the final results, determining the methods of presentation and publication, and selecting secondary projects and publications by the members of the steering committee. The Executive Committee also holds the right to modify or prematurely stop the study based on recommendations from the DSMB.

### Composition of the data monitoring committee, its role, and reporting structure {21a}

#### Data Safety Monitoring Board (DSMB)

The composition of the DSMB consists of a total of three members, including at least two cardiovascular intervention experts. The frequency of DSMB meetings will be determined prior to the study commencement. Additionally, the DSMB may convene a meeting at any time if there is a reason to suspect a safety issue. The DSMB is responsible for making recommendations regarding any safety or compliance issues throughout the course of the study and may recommend modifying or stopping the study to the Executive Committee. However, all final decisions regarding study modifications will be made by the Executive Committee. All cumulative safety data will be reported to the DSMB and reviewed on an ongoing basis throughout the enrollment and follow-up periods. Every effort will be made to allow the DSMB to conduct an unbiased review of patient safety information. All DSMB reports will be made available to the appropriate agencies or regulatory body upon request but will otherwise remain strictly confidential. Prior to the DSMB’s first review of the data, a DSMB charter will be drafted. The DSMB will develop a consensus understanding of all trial endpoints and definitions used in the event of an adjudication process.

### Adverse event reporting and harms {22}

#### Clinical Event Adjudication Committee (CEAC)

The Clinical Events Committee (CEAC) consists of interventional and non-interventional cardiologists who are not involved in the study. The CEAC is responsible for developing specific criteria for categorizing clinical events and clinical endpoints in the study based on the protocol. At the beginning of the trial, the CEAC will establish explicit rules outlining the minimum amount of data required and the algorithm to classify a clinical event. All CEAC members will be blinded to the primary results of the trial. The CEAC will meet regularly to review and adjudicate all clinical events and review and adjudicate all deaths that occur during the trial.

### Frequency and plans for auditing trial conduct {23}

Monthly trial steering committee meetings managed by the chairman will be held to maintain constant communication among the researchers and audit the trial conduct. Therefore, auditing trial conduct will be held regularly on a monthly basis, and the DSMB can convene meetings and audits at any time if they determine that there are safety issues.

### Plans for communicating important protocol amendments to relevant parties (e.g., trial participants, ethical committees) {25}

If there are changes to the protocol, the sponsor and funding agency will be notified first, and the principal investigator (PI) will inform the centers and send a copy of the revised protocol to the PI to be added to the investigator site file. It should be specified that any deviations from the protocol can be fully documented using a violation report form. The protocol will be updated in each clinical trial registry. Important protocol amendments will be communicated to trial participants, the ethics committee, and relevant parties in accordance with regulatory guidelines.

### Dissemination plans {31a}

A summary of the overall research process and findings will be distributed to each participating center to share with all study participants. In addition, trial results will be disseminated through publications in peer-reviewed journals, conference presentations, and clinical trial registries to ensure broad accessibility and transparency.

## Discussion

Coronary calcified lesions increase the complexity of PCI and are associated with adverse clinical outcomes [16, 17]. Severely calcified coronary lesions can impede lesion crossing, hinder appropriate stent expansion, damage the drug-eluting polymer, and increase the risk of stent-related complications, such as thrombosis and restenosis [18]. Thus, pretreatment of calcified lesions is important for improving prognosis, and various devices such as different types of balloons and arthrotome devices have been used for plaque modification [19].

The Wolverine™ balloon (Boston Scientific) is a novel cutting balloon mounted on a semi-compliant balloon with three or four arthrotomes bonded longitudinally to its surface [20]. Some studies been previously compared the use of cutting balloons with that of NC balloons in the treatment of coronary artery diseases; however, most of them focused on moderately or severely calcified lesions [16]. Another limitation of previous studies is the inclusion of a small number of patients and retrospective study design [11, 21]. Moreover, previous studies have mainly focused on periprocedural outcomes based on imaging studies, such as acute luminal gain and CSA, using IVUS [22, 23].

To achieve optimal preconditioning for stent implantation through active calcium modification, the indication for a cutting balloon in this study is not limited to severely calcified coronary artery disease but expanded to mildly calcified coronary artery disease, including ISR. For optimal preconditioning, balloon pre-dilatation and the following techniques will be performed for cutting balloon inflation: (1) nominal pressure with a balloon-to-vessel ratio of 1:1 or (2) high pressure using a cutting balloon downsized by 0.5 mm compared with the vessel’s media-to-media diameter. Accordingly, we aim to compare the procedural results between cutting and NC balloons through active calcium modification by comparing the lumen areas using QCA and IVUS.

In addition to the acute phase per-procedural gain of calcium modification, we aim to investigate long-term endpoints such as MACEs with aggressive calcium modification and an appropriate type of drug-eluting stent implantation. This trial will be conducted using a bioabsorbable polymer-coated, drug-eluting coronary stent. The SYNERGY stent (Boston Scientific Corporation) is a thin strut (74 μm) of platinum-chromium metal alloy that elutes everolimus from an ultrathin (4 μm) bioabsorbable polymer applied only to the abluminal surface of the stent and resolves within 4 months. After the absorption period of the polymer, only ultrathin (< 70 or 80 μm) stent struts remain, which can improve clinical outcomes in the treatment of various coronary artery diseases [24–26].

Studies have previously reported the stent crossability of novel cutting balloons for the treatment of calcium lesions. Periprocedural and intraprocedural factors, such as the amount of contrast medium and procedure time, are also associated with adverse clinical outcomes in the complex PCI era [7, 8]. Nevertheless, studies focusing on intraprocedural operator-centric factors, such as procedure duration, amount of contrast medium, and number of balloons used, are lacking [23]. In addition, as Amemiya et al. reported [27], the low crossability of the old model cutting balloon makes the operator hesitant to choose a cutting balloon as a first-line option for the treatment of calcified lesions. However, the crossability of the novel cutting balloon (Wolverine™) has been improved and proven to have a higher delivery success rate owing to the induction of superior crossability derived from active calcium modification [23]. Furthermore, owing to the development of balloon platform technologies, there have been reports on the improvement of stent and balloon crossability in the treatment of calcium lesions using novel cutting balloons compared with using old cutting or scoring balloons [23]. In our study, we focused on the intraprocedural and operator-centric outcomes, and the total procedure time, number of balloons, and amount of contrast medium used are set as secondary endpoint parameters.

In conclusion, in this prospective multicenter randomized open-label trial, we will evaluate the efficacy and safety of the novel cutting balloon (Wolverine™) and NC balloon in patients with calcified coronary lesions undergoing PCI and provide a rationale for which type of balloons will optimally modify calcium lesions. In addition, we will attempt to expand the indications of the cutting balloon for the treatment of mild-to-severe calcified coronary lesions by conducting a comparative study of NC and cutting balloons for the treatment of calcified lesions. We aim to investigate the clinical benefits of intraprocedural and periprocedural outcomes and operator convenience, including the total number of balloons used, procedure duration, and the amount of contrast medium used. Moreover, we aim to confirm the mid-to-long-term clinical benefits of aggressive modification of calcified lesions by cutting balloons, followed by the implantation of bioabsorbable polymer-coated everolimus-eluting coronary stents (Synergy™, Boston Scientific Corporation). As the scope of insurance coverage for cutting balloons remains limited in some countries, this study can provide evidence for extending insurance coverage to the treatment of de novo calcified and ISR lesions [28].

## Trial status

Protocol version 1.0 dated November 20, 2023. The first patient is planned to be randomized in January 2024, and the last patient is planned to be enrolled in December 2024.

## Data Availability

Data cannot be shared publicly owing to ethical and privacy concerns. Data are available from the Soonchunhyang University Bucheon Hospital Institutional Data Access/Ethics Committee (contact via the corresponding author) to researchers who meet the criteria for access to confidential data.

## Abbreviations

CEAC: Clinical Events Adjudication Committee
CRF: Case report form
CSA: Cross-sectional area
DSMB: Data Safety Monitoring Board
ISR: In-stent restenosis
IVUS: Intravascular ultrasound
MACE: Major adverse cardiac event
NC: Non-compliant
PCI: Percutaneous coronary intervention
QCA: Quantitative coronary angiography
